# Supplementary material for: Pioneer and nonpioneer factor cooperation drives lineage specific chromatin opening
Source: Nat Commun. 2019 Aug 23;10:3807. doi: 10.1038/s41467-019-11791-9 (PMC6707328; doi:10.1038/s41467-019-11791-9)
Supplement: Supplementary file 1 — Supplementary Information [file 41467_2019_11791_MOESM1_ESM.pdf]

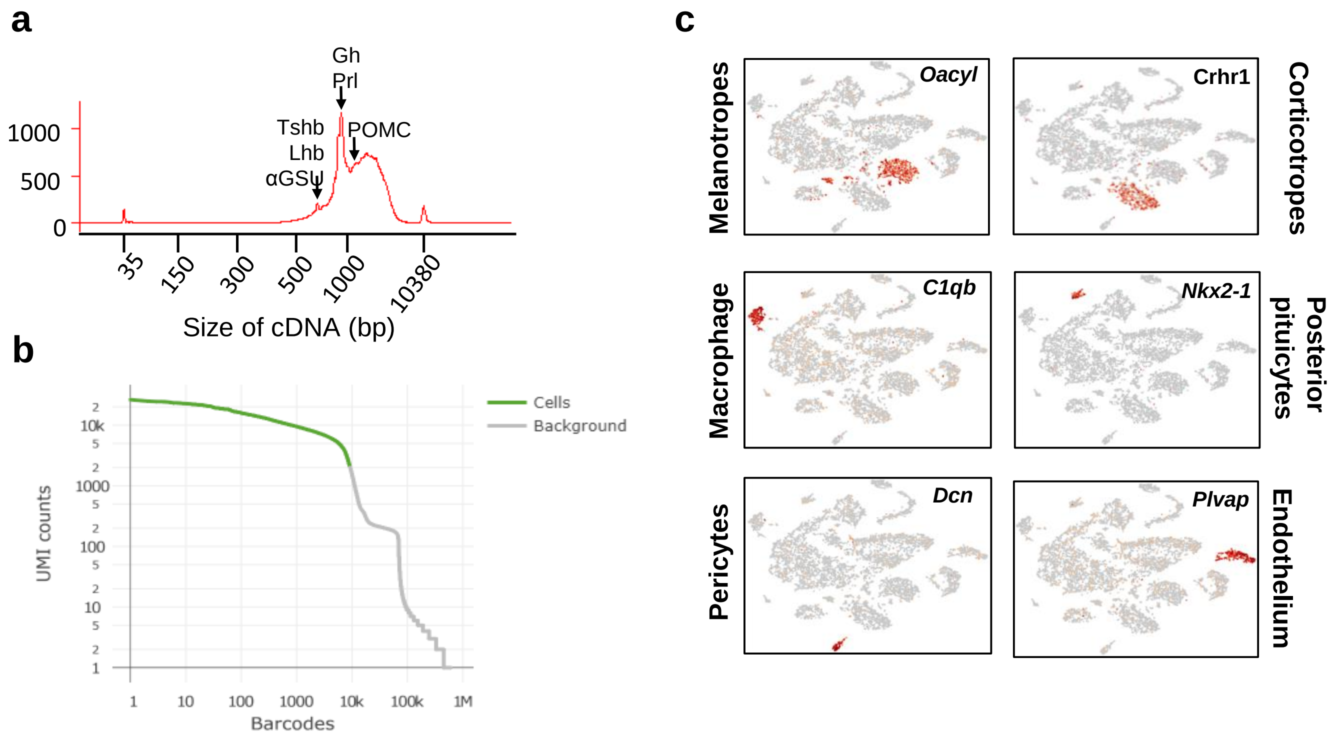

Supplementary Fig. 1. Validation of single cell RNAseq data.
a) Bioanalyzer profile showing cDNA size distribution from pituitary cells.
b) Single cell RNAseq plot showing barcode numbers (x-axis) over the number of UMI per barcode (y-axis). The threshold (colored) used for selecting the 9269 cells analysed.
c) t-SNE map showing color-coded expression of marker genes for the indicated lineages.

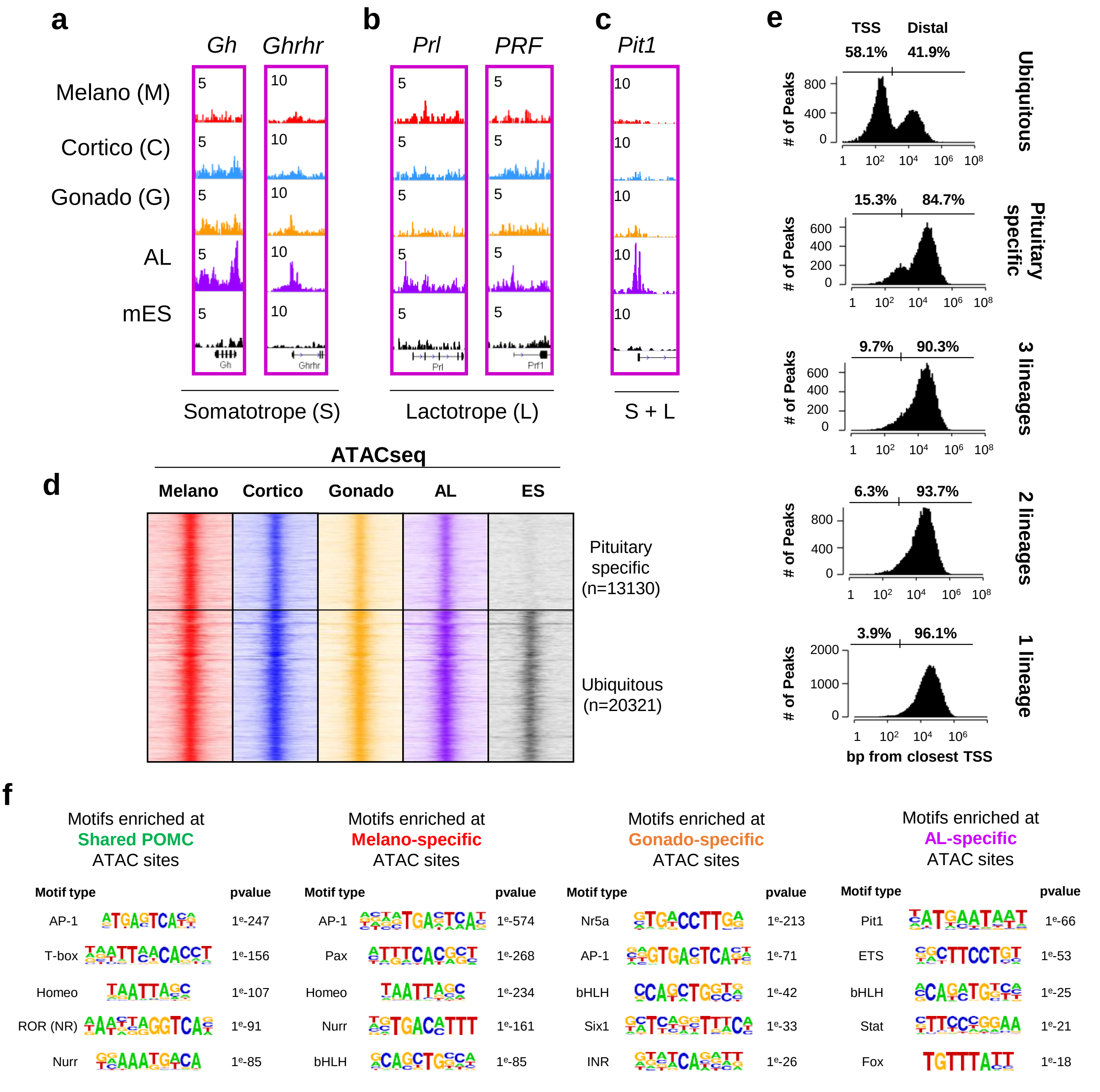

Supplementary Fig. 2. Chromatin landscapes in other pituitary lineages.

a-c) Genome browser views (IGV) of ATACseq profiles from the indicated pituitary lineages at genes marking the identity of somatotropes (A), lactotropes (B) and both (C).

d) Read density heatmaps showing ATACseq signals at sites of accessibility common to all four pituitary lineages clustered by ATACseq signals in mouse embryonic stem cells (GSE64058).

e) Genomic distribution of the distances between the indicated category of ATACseq peaks and the closest TSS.

f*) De novo* motif enrichment analyses (using HOMER) for the indicated subsets of ATACseq peaks. Motifs are ranked based on their P values; the top 5 motifs of each list are shown.

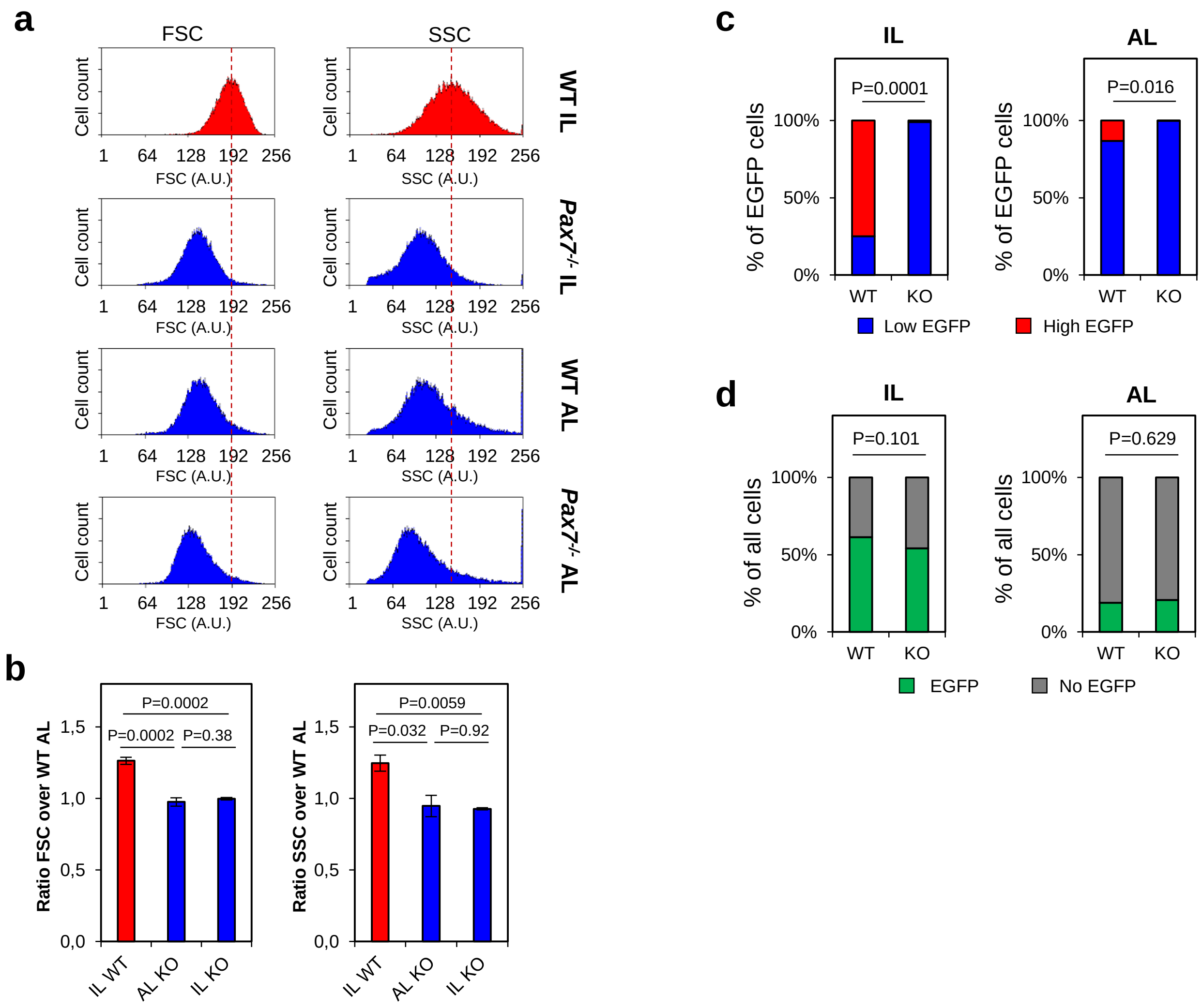

Supplementary Fig. 3. FACS analyses of wild-type (*WT*) and *Pax7-/- POMC-EGFP* pituitary cells.

a) Representative FACS profiles showing forward (left) and side (right) scatter for cells from wild type and *Pax7*$^{-/-}$ IL and AL.

b) Bar graphs showing the ratios of forward (right) and side (right) scatter for EGFP positive cells from wild type (n=5) and *Pax7*$^{-/-}$ (n=3) IL and AL.

c) Bar graph showing the proportion of high versus low EGFP expressing cells based on fluorescence signals detected by FACS. P values were computed using unpaired two-sided t-test.

d) Bar graph showing the proportion of EGFP expressing and non-expressing cells based on fluorescence signals detected by FACS. P values were computed using unpaired two-sided t-test.

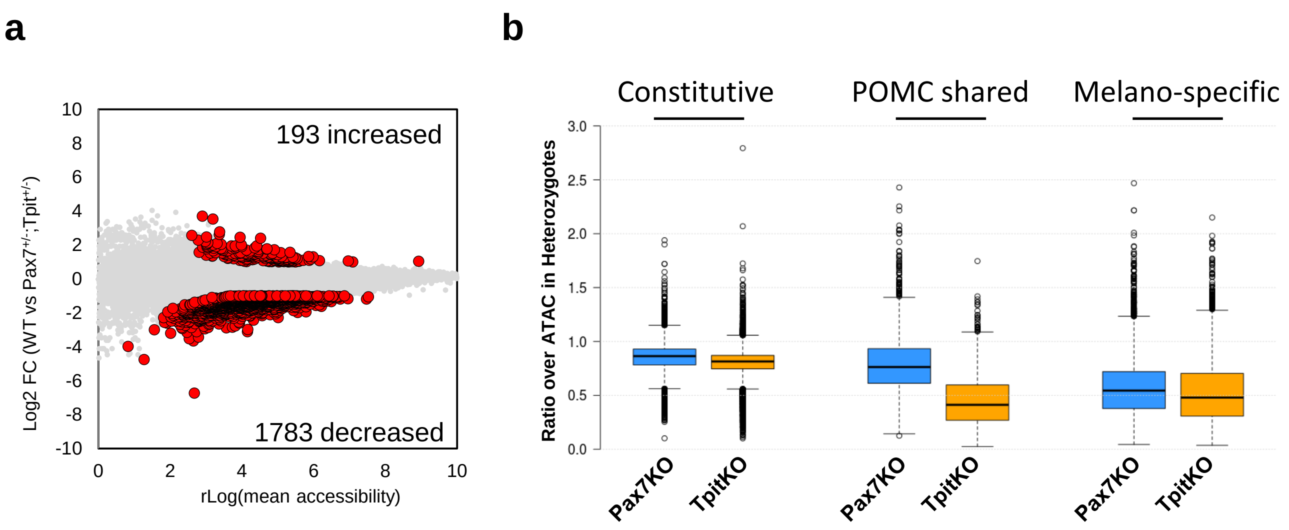

Supplementary Fig. 4. Comparison of ATACseq profiles in *WT* and *Pax7+/-;Tpit+/-* IL cells.

a) Dispersion plot showing rLog values of accessibility (ATACseq, x-axis) in wild type and *Pax7*$^{+/-}$;*Tpit*$^{+/-}$ IL over log2 fold changes in wild type versus *Pax7*$^{+/}$;*Tpit*$^{+/-}$ IL. Red circles identify the differentially accessible regions (p value<0.05, Log2 FC >+/-1).

b) Boxplots showing the ratios of ATACseq densities (RPKM) of *Pax7*$^{-/-}$;*Tpit*$^{+/-}$ (Pax7KO, blue) and *Pax7*$^{+/-}$;*Tpit*$^{-/-}$ (Tpit KO, yellow) over the *Pax7*$^{+/-}$;*Tpit*$^{+/-}$ (heterozygous) controls at the indicated subsets of ATACseq peaks The strongest peaks of each category were used (RPKM >2) to limit threshold effects. Center lines show medians; box limits indicate the twenty-fifth and seventy-fifth percentiles; whiskers extend to 1.5 times the interquartile range from the twenty-fifth to seventy-fifth percentiles. Outliers are shown by the individual circles.

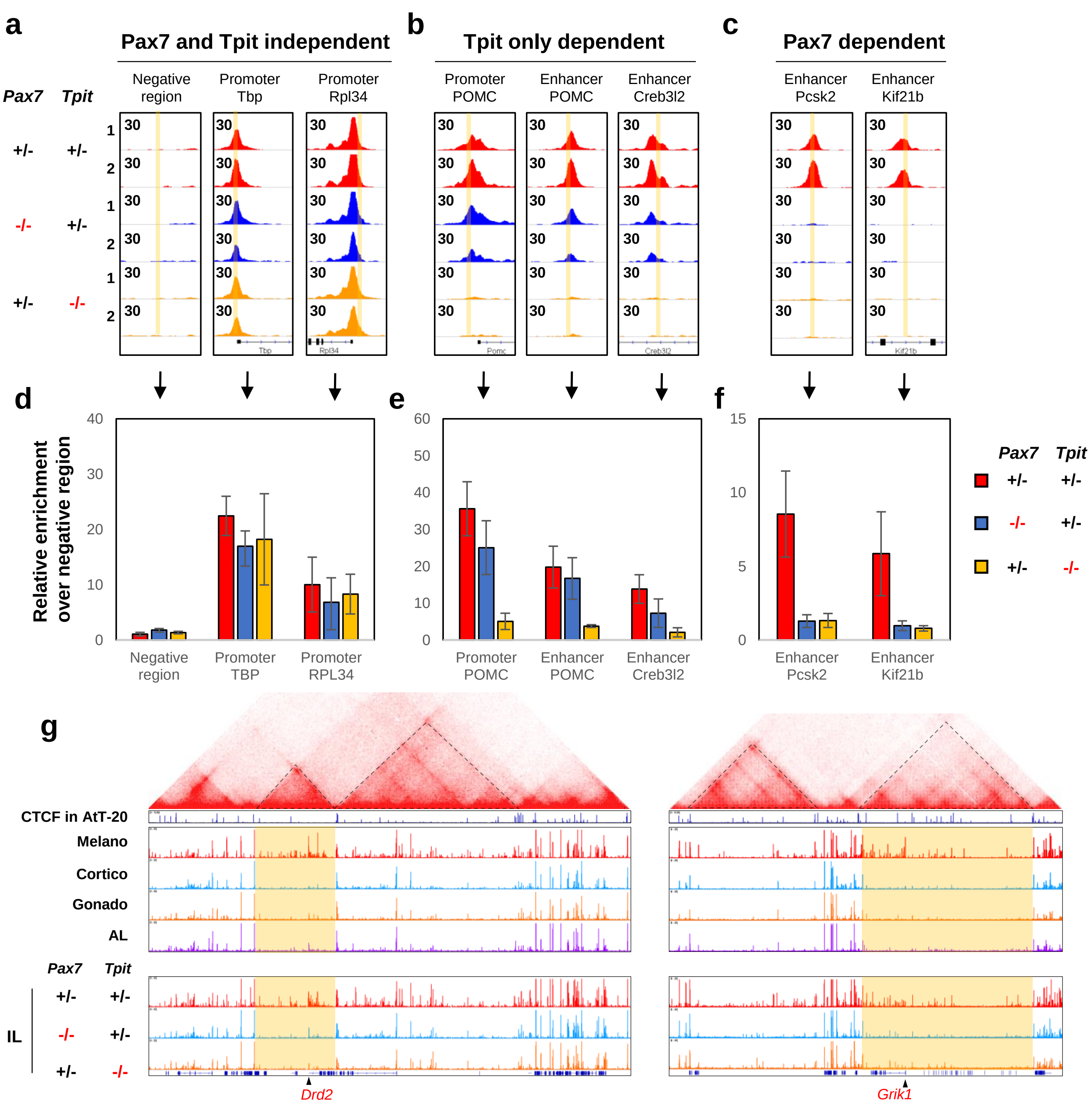

Supplementary Fig. 5. Q-PCR validation of Pax7 and/or Tpit-dependent accessibility (ATACseq).

a-c) Genome browser views (IGV) of ATACseq profiles from the indicated genotypes at unaffected (A), Tpit-only dependent (B) and Pax7 dependent (C) sites. Regions amplified in the qPCR measurements of Figure S5D-F are highlighted in yellow.

d-f) Relative enrichments over a negative region, measured by qPCR of ATACseq libraries at unaffected (D), Tpit-only dependent (E) and Pax7 dependent (F) sites.

g) Hi-C interaction map (top) from mouse ES cells (Bonev et al., 2017) around the *Drd2 (left)* and *Grik1 (right)* loci showing the boundaries of their respective TADs. Genome browser views (bottom) of the ATACseq profiles in purified pituitary cells and ILs of the indicated genotypes at the corresponding genome location. Arrowheads indicate gene promoters.

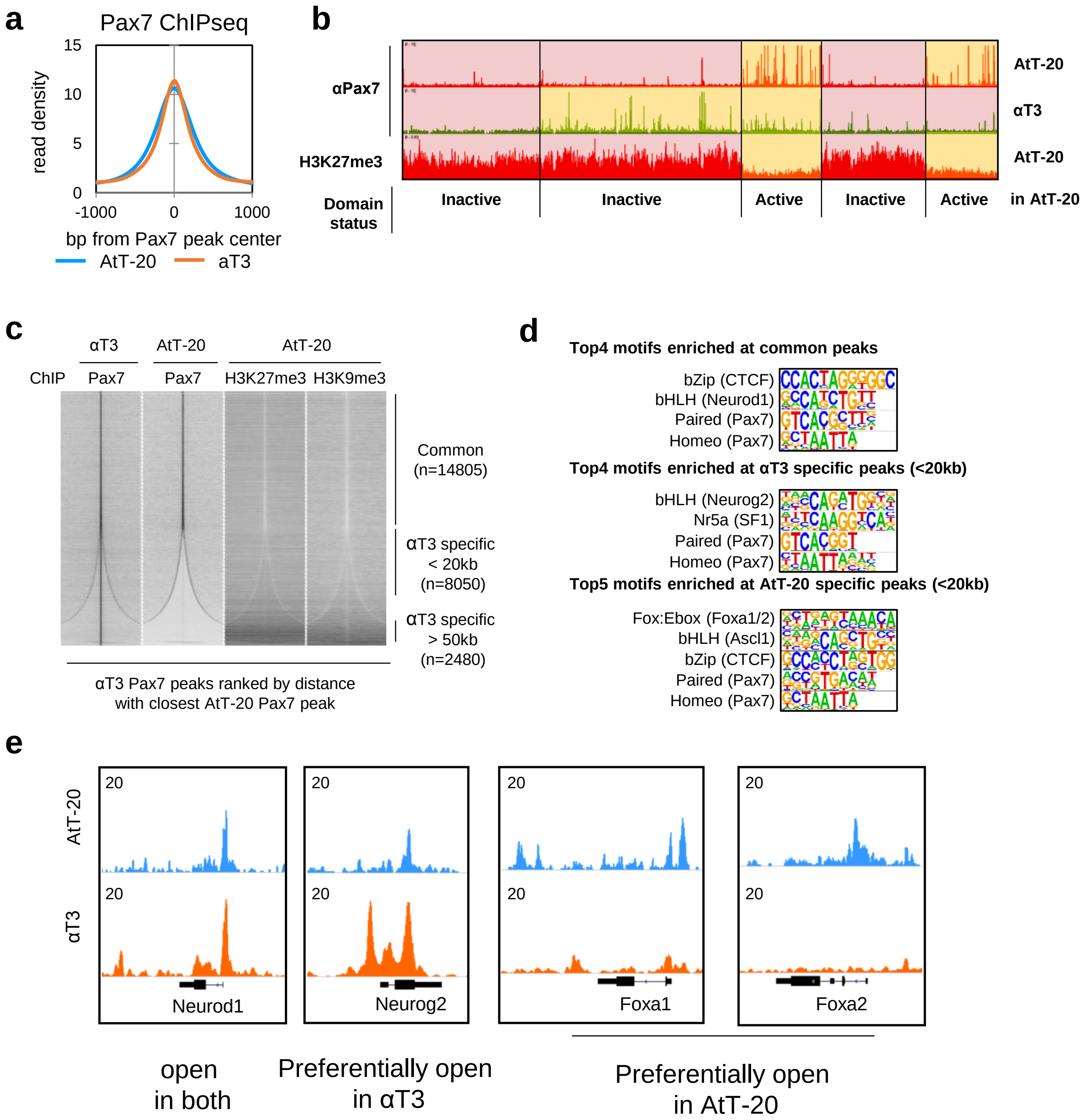

Supplementary Fig. 6. Comparison of Pax7 ChIPseq data in AtT-20 and αT3 cells.
a) Average profiles of Pax7 ChIPseq in AtT-20 (blue) and αT3 (orange) cells at the best 2000 Pax7 peaks from each lineage showing similar enrichments in both ChIPseqs.
b) Genome browser views (IGV) of Pax7 recruitment in AtT-20 and αT3 cells together with the H3K27me3 profile in AtT-20 cells. Pax7-bound (active) domains are shown in yellow, unbound (inactive) domains are shown in red.
c) Density heatmaps of Pax7 peaks in αT3 cells sorted by increasing distance to the closest Pax7 peak in AtT-20 cells. Windows (100kb wide) show Pax7 recruitment in αT3 and AtT-20 cells, together with H3K27me3 and H3K9me3 in AtT-20 cells.
d) *De novo* motif enrichment analyses (using HOMER) at the indicated subsets of Pax7 peaks. Motifs are ranked based on their P values.
e) Genome browser views of ATACseq profiles in αT3 and AtT-20 cells at the indicated loci.

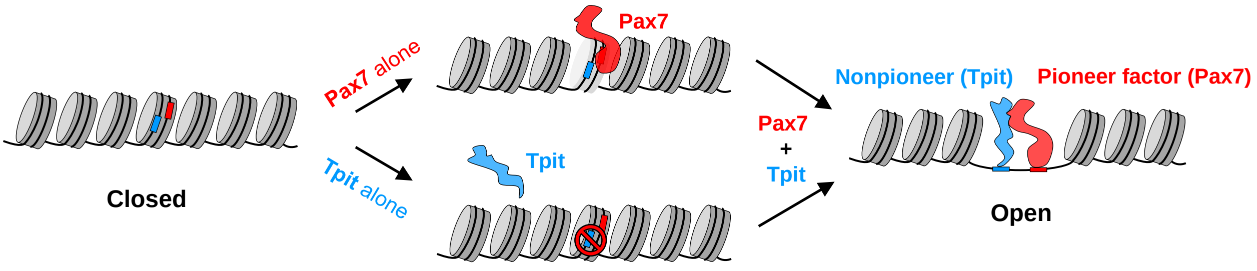

Supplementary Fig. 7. Proposed model of cooperative chromatin opening by pioneer and non-pioneer factors.
The heterochromatin target sites of Pax7 pioneer action in the pituitary typically contain Pax7 DNA binding sites together with Tpit binding sites within a few hundred bp of each other. These sites do not harbor any mark of active chromatin or present with a signal in ATACseq. These sites cannot be bound by Tpit on its own and upon Pax7 binding, they show weak ATACseq signal suggestive of an altered chromatin organisation. Recruitment of the nonpioneer factor Tpit at these sites involves Tpit interaction with its DNA binding site and protein interactions between Tpit and Pax7: these sites exhibit all the all marks of active enhancers, namely presence of H3K4me1, H3K27ac, p300, nucleosome depletion and ATAC sensitivity.
