# Supplementary material for: Pioneer and nonpioneer factor cooperation drives lineage specific chromatin opening
Source: Nat Commun. 2019 Aug 23;10:3807. doi: 10.1038/s41467-019-11791-9 (PMC6707328; doi:10.1038/s41467-019-11791-9)
Supplement: Supplementary file 2 — Reporting Summary [file 41467_2019_11791_MOESM2_ESM.pdf]

nature research

Corresponding author(s): Drouin

Last updated by author(s): Jun 26, 2019

Reporting Summary

Nature Research wishes to improve the reproducibility of the work that we publish. This form provides structure for consistency and transparency in reporting. For further information on Nature Research policies, see Authors & Referees and the Editorial Policy Checklist.

Statistics

For all statistical analyses, confirm that the following items are present in the figure legend, table legend, main text, or Methods section.

n/a	Confirmed	
☐	☒	The exact sample size (n) for each experimental group/condition, given as a discrete number and unit of measurement
☐	☒	A statement on whether measurements were taken from distinct samples or whether the same sample was measured repeatedly
☐	☒	The statistical test(s) used AND whether they are one- or two-sided<br>*Only common tests should be described solely by name; describe more complex techniques in the Methods section.*
☒	☐	A description of all covariates tested
☒	☐	A description of any assumptions or corrections, such as tests of normality and adjustment for multiple comparisons
☐	☒	A full description of the statistical parameters including central tendency (e.g. means) or other basic estimates (e.g. regression coefficient) AND variation (e.g. standard deviation) or associated estimates of uncertainty (e.g. confidence intervals)
☐	☒	For null hypothesis testing, the test statistic (e.g. F, t, r) with confidence intervals, effect sizes, degrees of freedom and P value noted<br>*Give P values as exact values whenever suitable.*
☒	☐	For Bayesian analysis, information on the choice of priors and Markov chain Monte Carlo settings
☒	☐	For hierarchical and complex designs, identification of the appropriate level for tests and full reporting of outcomes
☐	☒	Estimates of effect sizes (e.g. Cohen's d, Pearson's r), indicating how they were calculated

Our web collection on statistics for biologists contains articles on many of the points above.

Software and code

Policy information about availability of computer code

Data collection	Sequencing data collected by Illumina HiSeq4000 softwares.
Data analysis	Easeq1.02, HOMER v4.9.1, MACS v2.1.1.20160309, Bowtie v.2.3.1, Deseq2, R_Bioconductor v3.5.1_3.7, CellRangerv2.2, Summit (FACS analyses).

For manuscripts utilizing custom algorithms or software that are central to the research but not yet described in published literature, software must be made available to editors/reviewers. We strongly encourage code deposition in a community repository (e.g. GitHub). See the Nature Research guidelines for submitting code & software for further information.

Data

Policy information about availability of data

All manuscripts must include a data availability statement. This statement should provide the following information, where applicable:

- Accession codes, unique identifiers, or web links for publicly available datasets
- A list of figures that have associated raw data
- A description of any restrictions on data availability

All the sequencing data generated in this work are deposited on GEO as GSE125671.

Field-specific reporting

Please select the one below that is the best fit for your research. If you are not sure, read the appropriate sections before making your selection.

☒ Life sciences ☐ Behavioural & social sciences ☐ Ecological, evolutionary & environmental sciences

For a reference copy of the document with all sections, see nature.com/documents/nr-reporting-summary-flat.pdf

Life sciences study design

All studies must disclose on these points even when the disclosure is negative.

Sample size	No statistical methods were used to predetermine sample size
Data exclusions	There was no exclusion/inclusion of samples or animals in the analysis
Replication	FACS analyses of POMC-EGFP pituitary cells were performed on 5 WT and 3 KO replicates.<br>ATACseq on tissues of various genotypes were performed in duplicates (four lobes per replicate).<br>ATACseq on FACS-purified gonadotrope cells were performed once using 62 pituitaries.<br>ChIPseq experiments were performed once.<br>scRNAseq was performed using an adult male pituitary.
Randomization	There was no randomization of experiments
Blinding	Investigators were not blinded during experiments and analysis. Genotyping was done before performing the experiments.

Reporting for specific materials, systems and methods

We require information from authors about some types of materials, experimental systems and methods used in many studies. Here, indicate whether each material, system or method listed is relevant to your study. If you are not sure if a list item applies to your research, read the appropriate section before selecting a response.

Materials & experimental systems

n/a	Involved in the study
☐	☒ Antibodies
☐	☒ Eukaryotic cell lines
☒	☐ Palaeontology
☐	☒ Animals and other organisms
☒	☐ Human research participants
☒	☐ Clinical data

Methods

n/a	Involved in the study
☐	☒ ChIP-seq
☐	☒ Flow cytometry
☒	☐ MRI-based neuroimaging

Antibodies

Antibodies used	Listed in ChIPseq section.
Validation	*Describe the validation of each primary antibody for the species and application, noting any validation statements on the manufacturer's website, relevant citations, antibody profiles in online databases, or data provided in the manuscript.*

Eukaryotic cell lines

Policy information about cell lines

Cell line source(s)	AtT-20 cells obtained from the late E. Herbert in 1981 and maintained in this lab since.
Authentication	Cells were not authenticated by karyotyping but are routinely assessed for POMC expression and responsiveness to CRH and glucocorticoids, the hallmarks biological activities of corticotrope cells that constitute the basis for using this model.
Mycoplasma contamination	Yes, on a yearly basis.
Commonly misidentified lines<br>(See ICLAC register)	No commonly misidentified cell lines were used in this study.

Animals and other organisms

Policy information about studies involving animals; ARRIVE guidelines recommended for reporting animal research

Laboratory animals	Stated in Online Methods, mouse strains used are WT or POMC-EGFP (C57Bl/6), and Pax7-/- (129/sv) and Tpit-/- (Balb/c).
Wild animals	Not used in this study.

Field-collected samples	Not used in this study.
Ethics oversight	Animal studies were approved by the IRCM Animal Ethics Committee following Canadian regulations.

Note that full information on the approval of the study protocol must also be provided in the manuscript.

ChIP-seq

Data deposition

☒ Confirm that both raw and final processed data have been deposited in a public database such as GEO.

☒ Confirm that you have deposited or provided access to graph files (e.g. BED files) for the called peaks.

Data access links
May remain private before publication.

GEO GSE125671

Files in database submission

(1) GSE125668: Pioneer and nonpioneer factor cooperation drives lineage specific chromatin opening [ATAC-seq]
GSM3579919
ATACseq in purified anterior pituitary
GSM3579920
ATACseq in purified gonadotropes
GSM3579921
ATACseq in wild type intermediate pituitary rep1
GSM3579922
ATACseq in wild type intermediate pituitary rep2
GSM3579923
ATACseq in Pax7 Het;Tpit Het intermediate pituitary rep1
GSM3579924
ATACseq in Pax7 Het;Tpit Het intermediate pituitary rep2
GSM3579925
ATACseq in Pax7 Knockout intermediate pituitary rep1
GSM3579926
ATACseq in Pax7 Knockout intermediate pituitary rep2
GSM3579927
ATACseq in Tpit Knockout intermediate pituitary rep1
GSM3579928
ATACseq in Tpit Knockout intermediate pituitary rep2
GSM3579929
ATACseq in Pax7 and Tpit double Knockout intermediate pituitary rep1
GSM3579930
ATACseq in αT3 cells expressing Neo
GSM3579931
ATACseq in αT3 cells expressing Pax7
GSM3579932
ATACseq in AtT-20 cells expressing Neo replicate 1
GSM3579933
ATACseq in AtT-20 cells expressing Neo replicate 2
GSM3579934
ATACseq in AtT-20 cells expressing Pax7 replicate 1
GSM3579935
ATACseq in AtT-20 cells expressing Pax7 replicate 2

(2) GSE125669: Pioneer and nonpioneer factor cooperation drives lineage specific chromatin opening [ChIP-seq]
GSM3579936
Pax7 (Flag) ChIPseq in αT3 cells expressing Pax7
GSM3579937
Flag (control) ChIPseq in αT3 cells expressing Neo
GSM3579938
Input DNA in αT3 cells expressing Pax7
GSM3579939
Input DNA in αT3 cells expressing Neo
GSM3579940
Neurod1 ChIPseq in AtT-20 cells
GSM3579941
Sox2 ChIPseq in AtT-20 cells expressing Sox2

(3) GSE125670: Pioneer and nonpioneer factor cooperation drives lineage specific chromatin opening [scRNA-seq]
GSM3579942Single cell RNAseq from adult male pituitary

Genome browser session
(e.g. UCSC)

N/A

Methodology

Replicates	ATACseq on tissues of various genotypes were performed in duplicates (four lobes per replicate).<br>ATACseq on FACS-purified gonadotrope cells were performed once using 62 pituitaries.<br>ChIPseq experiments were performed once.
Sequencing depth	ATACseq in sorted gonadotropes: 45 641 768 reads PE50<br>ATACseq in sorted anterior lobe : 56 366 675 reads PE50<br>ATACseq in Pax7+/+;Tpit+/+ IL rep1: 27 878 000 reads PE125<br>ATACseq in Pax7+/+;Tpit+/+ IL rep2: 27 219 724 reads PE125<br>ATACseq in Pax7+/-;Tpit+/- IL rep1: 35 018 234 reads PE125<br>ATACseq in Pax7+/-;Tpit+/- IL rep2: 76 601 522 reads PE125<br>ATACseq in Pax7-/-;Tpit+/- IL rep1: 25 998 246 reads PE125<br>ATACseq in Pax7-/-;Tpit+/- IL rep2: 59 145 908 reads PE125<br>ATACseq in Pax7+/-;Tpit-/- IL rep1: 38 130 840 reads PE125<br>ATACseq in Pax7+/-;Tpit-/- IL rep2: 61 087 617 reads PE125<br>ATACseq in Pax7-/-;Tpit-/- IL rep1: 60 155 847 reads PE125<br>ATAC in AtT-20 Neo rep1: 23 668 280 reads PE125<br>ATAC in AtT-20 Neo rep: 31 075 766reads PE125<br>ATAC in AtT-20 Pax7 rep1: 21 809 982 reads PE125<br>ATAC in AtT-20 Pax7 rep2: 33 953 754 reads PE125<br>ATAC in aT3 Neo rep1: 48 752 854reads PE50<br>ATAC in aT3 Pax7 rep1: 58 172 820 reads PE50<br>Sox2 ChIPseq in AtT-20: 59 166 753 reads PE50<br>Neurod1 ChIPseq in AtT-20: 59 624 919 reads PE50<br>Pax7 (Flag) ChIPseq in aT3 Pax7: 48 881 695 reads PE50<br>Flag ChIPseq in aT3 Neo: 32 560 399 reads PE50
Antibodies	Sox2: Ab59776 Abcam<br>Flag: F3165 Sigma<br>Neurod1: Poulin et al. 2000
Peak calling parameters	Peaks were identified by comparing each sample to its input using MACS v2.1.1.20160309 callpeak function using the parameters: --bw 250 -g mm --mfold 10 30 -p 1e-5.
Data quality	ATACseq in sorted gonadotropes: 66 140 peaks with pvalue<0.00005 and 46 554 with Fold >5<br>ATACseq in sorted anterior lobe : 59 116 peaks with pvalue<0.00005 and 37 383 with Fold >5<br>ATACseq in Pax7+/+;Tpit+/+ IL rep1: 39 025 peaks with pvalue<0.00005 and 36 014 with Fold >5<br>ATACseq in Pax7+/+;Tpit+/+ IL rep2: 49 348 peaks with pvalue<0.00005 and 45 851with Fold >5<br>ATACseq in Pax7+/-;Tpit+/- IL rep1: 55 466 peaks with pvalue<0.00005 and 50 318 with Fold >5<br>ATACseq in Pax7+/-;Tpit+/- IL rep2: 70 135 peaks with pvalue<0.00005 and 47 496 with Fold >5<br>ATACseq in Pax7-/-;Tpit+/- IL rep1: 21 028 peaks with pvalue<0.00005 and 17 847 with Fold >5<br>ATACseq in Pax7-/-;Tpit+/- IL rep2: 52 126 peaks with pvalue<0.00005 and 35 143 with Fold >5<br>ATACseq in Pax7+/-;Tpit-/- IL rep1: 33 895 peaks with pvalue<0.00005 and 25 732 with Fold >5<br>ATACseq in Pax7+/-;Tpit-/- IL rep2: 56 822 peaks with pvalue<0.00005 and 37 050 with Fold >5<br>ATACseq in Pax7-/-;Tpit-/- IL rep1: 46 388 peaks with pvalue<0.00005 and 31 209 with Fold >5<br>ATAC in aT3 Neo rep1: 59 161 peaks with pvalue<0.00005 and 47 818 with Fold >5<br>ATAC in aT3 Pax7 rep1: 68 732 peaks with pvalue<0.00005 and 49 254 with Fold >5<br>Sox2 ChIPseq in AtT-20: 57 762 peaks with pvalue<0.00005 and 38 099 with Fold >5<br>Neurod1 ChIPseq in AtT-20: 102 688 peaks with pvalue<0.00005 and 74 774 with Fold >5<br>Pax7 (Flag) ChIPseq in aT3 Pax7: 68 410 peaks with pvalue<0.00005 and 42 192 with Fold >5
Software	Easeq1.02, HOMER v4.9.1, MACS v2.1.1.20160309, Bowtie v.2.3.1, Deseq2, R_Bioconductor v3.5.1_3.7

Flow Cytometry

Plots

Confirm that:

- [x] The axis labels state the marker and fluorochrome used (e.g. CD4-FITC).
- [x] The axis scales are clearly visible. Include numbers along axes only for bottom left plot of group (a 'group' is an analysis of identical markers).
- [ ] All plots are contour plots with outliers or pseudocolor plots.
- [ ] A numerical value for number of cells or percentage (with statistics) is provided.

Methodology

Sample preparation	62 anterior lobe from pituitary of Tg(LH-Cerulean) mice were dissociated (as described in methods) and sorted based on Cerulean signal for the purification of Pituitary lineages.

	Single anterior or intermediate lobe of Tg(POMC-EGFP);Pax7+/- or Tg(POMC-EGFP);Pax7-/-were dissociated (as described in methods) for FACS analysis
Instrument	FACSAria (BD) was used for Purification of pituitary lineage<br>FACSCalibur (BD) was used for FACS analyses
Software	Analysis of FACS data was performed using Summit
Cell population abundance	Sorting was based on the Cerulean level that strongly marks gonadotropes in Tg(LH-Cerulean), the population of interest, WT mice were used to identify background level of Cerulean signal
Gating strategy	FSC and SSC gates were set to avoid debris and doublets of cells. Cerulean signal was used to differentiate the gonadotropes and the non-gonadotrope cells of anterior pituitaries

☐ Tick this box to confirm that a figure exemplifying the gating strategy is provided in the Supplementary Information.
